# Articles selected from posters presented at the Tenth Annual International Conference on Research in Computational Biology – Preface

**DOI:** 10.1186/1471-2105-8-S5-S1

**Published:** 2007-05-24

**Authors:** Alberto Apostolico, Raffaele Giancarlo, Concettina Guerra, Giuseppe Lancia

**Affiliations:** 1 College of Computing, Georgia Tech, GA, USA; 2 Dipartimento di Ingegneria Informatica, Universitá di Padova, Italy; 3 Dipartimento di Matematica ed Applicazioni, Universitá di Palermo, Italy; 4 Dipartimento di Matematica ed Informatica, Universitá di Udine, Italy

The synergies among biology, computing and other formal disciplines continue to produce a unique blend of domain-specific and methodological advances that is shaping the very fabrics of the new scientific method. Among the many examples of this phenomenon, the one offered by the unrelenting growth of bioinformatics and computational biology is unique in that nowhere else is the native lexicon of a natural science more directly conducive to digital representation and manipulation.

The research articles contained in this Supplement originate from posters presented at the *Tenth Annual International Conference on Research in Computational Molecular Biology *(RECOMB 2006), which was held in Venice, Italy, on April 2–5, 2006. The RECOMB conference series was started in 1997 by Sorin Istrail, Pavel Pevzner and Michael Waterman. The previous meetings were held in Santa Fe, NM (USA); New York, NY (USA); Lyon, France; Tokyo, Japan; Montréal, Canada; Washington, DC (USA); Berlin, Germany; San Diego, CA (USA); and Boston, MA (USA). RECOMB 2006 was hosted by University of Padova in the Venice Convention Center at Cinema Palace, Venice Lido, Italy.

The Tenth Edition of RECOMB was special in several ways. For one, the Program Committee, consisting of 38 specialists of the highest distinction in the field, included all past Committee and Conference Chairs as well as the Members of the Steering Committee. The Committee selected 40 papers out of the received submissions of well over 200. Some of the accepted papers were further expanded and refereed in order to appear in the special issue traditionally devoted to the Conference. For the Tenth Edition, however, in view of the high quality of the contributions submitted for poster presentation, it was felt that another Special Issue, devoted to expanded and duly refereed versions of poster submissions was also warranted.

Thus, the present Supplement constitutes one more innovation brought about by the Tenth Anniversary of RECOMB. The eight enclosed papers emerged at the outset of a rigid selection and review and represent a vivid snapshot of work mature enough to be reported within vibrant frameworks still in the making. We hope that they will start one more tradition for RECOMB.

This Issue was made possible thanks to the effort of many, in particular, the special task force set up for handling posters, which consisted of Luca Bortolussi (University of Udine, Italy), Giovanni Ciriello (University of Padova, Italy), Matteo Comin (University of Padova, Italy), Claudio Garrutti (University of Padova, Italy), Giosué Lo Bosco (University of Palermo, Italy), Sabrina Mantaci (University of Palermo, Italy) Cinzia Pizzi (University of Padova, Italy, and Helsinki, Finland), Simone Scalabrin (University of Udine, Italy), and Nicola Vitacolonna (University of Udine, Italy).

We are also grateful to the external reviewers, the members of the Steering Committee and other colleagues who helped in the process. Finally, we express our thanks to the institutions and corporations who provided financial support for the conference: Broad Institute of MIT and Harvard, USA; College of Computing, Georgia Tech., USA; Department of Energy, USA; Department of Information Engineering, University of Padova, Italy; IBM Corporation, USA; ISMB, International Society for Computational Biology; AICA, Italian Association for Informatics and Automatic Computation; National Science Foundation, USA; University of Padova, Italy.

